# Metabolic syndrome is not associated with erosive hand osteoarthritis: a cross-sectional study using data from the PROCOAC cohort

**DOI:** 10.1038/s41598-024-55374-1

**Published:** 2024-03-12

**Authors:** Maite Silva-Díaz, Sonia Pértega-Díaz, Vanesa Balboa-Barreiro, Carlos M. Tilve-Álvarez, Ana Raga-Sivira, Ignacio Rego-Pérez, Francisco J. Blanco, Natividad Oreiro

**Affiliations:** 1 Grupo de Investigación Clinica en Reumatología (GIR), INIBIC-Complejo Hospitalario Universitario A Coruña (CHUAC), SERGAS, As Xubias, 15006 A Coruña, Spain; 2 Grupo de Investigación en Reumatología y Salud (GIR-S), Centro de Investigaciones Científicas Avanzadas (CICA), Universidade da Coruña, A Coruña, Spain; 3 Unidad de Epidemiología Clínica y Bioestadística, INIBIC-Complejo Hospitalario Universitario A Coruña (CHUAC), SERGAS, A Coruña, Spain; 4 Avances en Telemedicina e Informática Sanitaria (ATIS), INIBIC-Complejo Hospitalario Universitario A Coruña (CHUAC), SERGAS, A Coruña, Spain; 5 Grupo de Investigación en Reumatología (GIR), INIBIC-Complejo Hospitalario Universitario A Coruña (CHUAC), SERGAS, A Coruña, Spain

**Keywords:** Osteoarthritis, Metabolic syndrome, Risk, Hand osteoarthritis, Knee osteoarthritis, Chondrocytes, Rheumatology, Rheumatic diseases, Osteoarthritis

## Abstract

To delineate the phenotype of erosive hand osteoarthritis (EHOA) in a Spanish population and assess its correlation with metabolic syndrome. We conducted a cross-sectional study using baseline data from the Prospective Cohort of Osteoarthritis from A Coruña (PROCOAC). Demographic and clinical variables, obtained through questionnaires, clinical examinations, and patient analytics, were compared among individuals with hand OA, with and without EHOA. We performed appropriate univariate and multivariate stepwise regression analyses using SPSS v28. Among 1039 subjects diagnosed with hand OA, 303 exhibited EHOA. Multivariate logistic regression analysis revealed associations with inflamed joints, nodular hand OA, and total AUSCAN. Furthermore, the association with a lower prevalence of knee OA remained significant. The influence of metabolic syndrome (MetS) on EHOA patients was analyzed by including MetS as a covariate in the model. It was observed that MetS does not significantly impact the presence of EHOA, maintaining the effect size of other factors. In conclusion, in the PROCOAC cohort, EHOA is associated with nodular hand OA, inflammatory hand OA, and a higher total AUSCAN. However, EHOA is linked to a lower prevalence of knee OA. Importantly, in our cohort, no relationship was found between EHOA and MetS.

## Introduction

Osteoarthritis (OA) of the hand is a highly prevalent disease, notably exhibiting a recognized female predominance^1,2^. The prevalence estimates vary depending on the definition employed. Radiographic hand OA, associated with the highest prevalence (ranging from 29 to 76%), contrasts with the symptomatic type, where frequencies are lower (ranging from 3 to 16%)^1–3^. Furthermore, compared with other forms of OA, such as knee or hip, hand OA generally has a higher prevalence^4^.

Hand OA encompasses various subsets^5^ and is acknowledged as a heterogeneous disease resulting in considerable disability with variable manifestations^6–10^. Erosive hand OA (EHOA) stands out among these subsets. The term “erosive” was first coined in 1966 by Peter and colleagues^11^. However, Crain previously used the term “Interphalangeal osteoarthritis” to describe a localized form characterized by destructive changes, intermittent inflammatory episodes, and eventual deformities and ankyloses^12^. While EULAR recommendations consider EHOA as a subset of hand OA, controversy arises due to its unclear etiopathogenesis. There’s a debate on whether to regard EHOA as a distinct disease entity or a severe phase within the continuum of hand OA^13,14^. Data from the Framingham Osteoarthritis study revealed EHOA prevalence ranging from 3.6% in men to 9.8% in women, increasing to 9.5% and 24.3%, respectively, over 60 years. Its development mainly occurs in patients with non-erosive hand OA (non-EHOA) at baseline^15^.

EHOA is radiographically defined by subchondral bone erosions in interphalangeal joints, cortical destruction, and subsequent reparative changes, including potential bony ankylosis^5^. Clinical features involve an abrupt onset of severe pain, varying degrees of stiffness, joint swelling, deformities, and erythema^16,17^. Additionally, EHOA is characterized by more severe and frequent synovitis, as well as radiographic progression^18^.

Hand OA commonly occurs, although not exclusively, in the context of generalized OA^5^, but findings in this regard are somewhat conflicting^19–23^. Controversy also exists regarding the varying degree of association between metabolic alterations—such as dyslipidemia, diabetes, hypertension, or overweight—and hand OA or EHOA^24–28^. Based on this evidence, further investigation is warranted to determine whether metabolic disturbances, specifically the metabolic syndrome, are associated with severe hand OA and whether this association differs for EHOA compared to non-EHOA.

With this background, the aim of this study is to describe the EHOA phenotype in the PROCOAC cohort (Prospective Cohort of Osteoarthritis from A Coruña) and its relationship with metabolic syndrome (MetS).

## Methods

For this study, we utilized cross-sectional data at baseline (when the patient was diagnosed) from the PROCOAC cohort, a population-based study investigating the determinants and severity of osteoarthritis (OA) in the knees, hips, and hands^29^. The Galician ethics committee approved the study, and written informed consent was obtained from all participants. Patient recruitment commenced in 2006. Patients were included based on the following criteria: i) patients from Rheumatology consultations with hand pain and diagnosed with hand OA following ACR criteria; ii) patients with knee pain diagnosed with radiographic knee OA following ACR criteria; and iii) patients with hip pain diagnosed with radiographic hip OA following ACR criteria. The cohort comprised 1252 subjects, of which 1039 were diagnosed with hand OA following ACR criteria^30,31^.

### Statement of ethics and consent

The Galician ethics committee, part of Xunta de Galicia, Spain, approved the study, and written informed consent was obtained from all participants. Research was conducted in accordance with relevant guidelines/regulations, and the manuscript includes a statement confirming that informed consent was obtained from all participants and/or their legal guardians. Research involving human participants must have been performed following the Declaration of Helsinki.

### Measurements

All X-rays from patients with hand OA underwent rigorous review to confirm the erosive phenotype^31^, conducted by both a radiologist and a trained rheumatologist specialist in a blinded manner. Erosive hand OA (EHOA) was radiographically defined by subchondral erosion, cortical destruction, and subsequent reparative changes in interphalangeal (IP) joints, potentially involving bony ankylosis in at least one hand. Additionally, the total number of IP joints with erosions per patient was counted and recorded. The reliability of all X-rays was meticulously assessed. Evaluation of right and left-hand joints, including wrists, was conducted using anteroposterior images. Magnified views of the entire hand were employed to confirm the presence of typical erosions^4^.

For this study, baseline data included the following variables: age, gender, smoking habit (categorized as never smoker, current smoker, or former smoker), body mass index (BMI), abdominal perimeter, personal history of hypertension, type 2 diabetes, dyslipidemia, psoriasis, osteoporosis, and osteopenia. Definitions for conditions included systolic blood pressure ≥ 140 mmHg and/or diastolic blood pressure ≥ 90 mmHg for hypertension^32^, fasting plasma glucose ≥ 126 mg/dL, glycated hemoglobin ≥ 6.5%, or classic symptoms of hyperglycemia for diabetes^33^, total cholesterol ≥ 220 mg/dL, low-density lipoprotein (LDL) ≥ 150, high-density lipoprotein (HDL) < 40 mg/dL in men and < 50 mg/dL in women, and triglycerides > 150 mg/dL for dyslipidemia. Osteoporosis was defined as a bone mineral density (BMD) ≤ − 2.5 SD in the femoral neck or lumbar spine, and osteopenia as BMD between − 1.5 and − 2.5 SD^34^. Metabolic Syndrome status was assessed following ALAD (Asociación Latinoamericana de Diabetes) 2010 criteria^35^, considering criteria such as waist circumference ≥ 94 cm in men and ≥ 88 cm in women, and meeting at least 2 of 4 specified criteria. Nodular hand OA was defined as Heberden and/or Bouchard nodes plus underlying interphalangeal damage. Thumb base OA, inflamed IP joints (characterized by swelling, heat, pain, and redness), and fasting blood levels of glucose, LDL, HDL, triglycerides, erythrocyte sedimentation rate (ESR), C-reactive protein (CRP), rheumatoid factor (RF), and anticitrulline antibodies (ACPA) were also collected at baseline. Additionally, the AUStralian CANadian index (AUSCAN Osteoarthritis Hand index NRS 3.1) questionnaire was assessed in a subset of 575 hand OA patients, providing a total score that considers pain, stiffness, and function, with each section evaluated on a scale from 0 to 100^36,37^.

### Statistical analysis

The statistical analysis encompassed a descriptive examination of the studied variables. Continuous variables were presented as mean ± standard deviation (SD) and median, while qualitative variables were articulated in absolute values (n) and percentages.

Mean comparisons between two groups were conducted utilizing the Student’s t-test or Mann–Whitney test, depending on appropriateness, subsequent to normality verification through the Kolmogorov–Smirnov test. Associations of qualitative variables were scrutinized using the Pearson Chi-Square test.

Univariate and multivariate logistic regression models were employed to investigate factors linked to the presence of erosive osteoarthritis (OA) of the hand. Construction of the multivariable models adhered to the backward selection method, where the Wald statistic served as the elimination criterion in each step, retaining variables with a significance level of *p* < 0.05 or clinical relevance. The impact of MetS on the probability of presenting EHOA was assessed by adjusting for variables selected in the final model. The effect of each factor was expressed through estimation of the odds ratio (OR) and its 95% confidence interval (CI). The area under the receiver operating characteristic (ROC) curve (AUC) quantified the discrimination performance of the EHOA predictive models. Sensitivity, specificity, and predictive values were reported with their 95% confidence interval.

The relationship between age and the risk of EHOA was explored using multivariable-adjusted cubic splines curves, incorporating five knots in the model.

All analyses were conducted using statistical software, specifically SPSS v.28 and R v.4.2. The “pROC” package facilitated ROC analysis, while restricted cubic splines regression was implemented with the “rms” package. Significance levels reported were two-sided, and the threshold for statistical significance was defined as *p* < 0.05.

## Results

A total of 1039 patients had hand OA with a mean age of 62.95 years and a predominance of women (82.58%). Table 1 illustrates the characteristics of our study population. Within this cohort, 303 patients (29.16%) had EHOA.

**Table 1:** Descriptive analysis of hand OA on PROCOAC cohort.

	Mean (SD)	Median	n (%)
Age at diagnosis	62.95 (9.09)	63.00	
Sex			
Male			181 (17.42)
Female			858 (82.58)
BMI	29.68 (17.27)	28.60	
Abdominal perimeter	95.22 (12.32)	95.00	
Hypertension			499 (48.21)
Diabetes			186 (18.02)
Dyslipidemia			660 (58.67)
Hypertriglyceridemia			168 (16.80)
Low HDL			95 (9.46)
MetS			202 (19.55)
Osteoporosis			166 (15.98)
Osteopenia			107 (10.30)
Smoking habit			
Never			659 (63.73)
Current			253 (24.47)
Former			122 (11.80)
Psoriasis			87 (8.41)
Total AUSCAN	45.49 (29.35)	48.80	
Pain AUSCAN	47.32 (31.15)	50.40	
Stiffness AUSCAN	44.05 (35.71)	50.00	
Function AUSCAN	44.64 (30.34)	48.78	
Knee OA (KL > I)			713 (70.52)
Hip OA			515 (51.55)
EHOA			303 (29.16)
Inflammatory hand OA			117 (11.35)
Nodular hand OA			856 (83.84)
Thumb base OA			649 (63.26)
ESR	19.71 (14.47)	16.00	
CRP	0.39 (0.88)	0.21	
Positive RF			81 (8.67)
Positive ACPA			12 (1.39)

BMI, body mass index (Kg/m^2^); HDL, high-density lipoprotein; MetS, metabolic syndrome; Knee OA, OA at least in one knee; Hip OA, OA at least in one hip; EHOA, erosive hand osteoarthritis at least in one hand; Inflammatory hand OA, OA at least in one hand; Nodular hand OA, OA at least in one hand; Thumb base OA, OA at least in one hand; ESR, erythrocyte sedimentation rate (mmHg); CRP, C-reactive protein (mg/dL); RF, rheumatoid factor; ACPA, anti-citrullinated protein antibodies.

Patients with EHOA in the PROCOAC cohort were younger (59 ± 8.17 vs. 65 ± 8.97; *p* < 0.001) and had a lower abdominal perimeter (93 ± 12.71 vs. 96 ± 11.98; *p* = 0.002) than non-EHOA patients (Table 2). AUSCAN scores were significantly higher in the EHOA group than in the non-EHOA group, including total AUSCAN (54 ± 26.92 vs. 41 ± 29.50; *p* < 0.001), and all its components: pain (56 ± 27.81 vs. 43 ± 31.85; *p* < 0.001), stiffness (54 ± 32.88 vs. 39 ± 36; *p* < 0.001), and function (54 ± 28.44 vs. 40 ± 30.28; *p* < 0.001).

**Table 2:** Descriptive analysis and comparison of hand OA patients according to EHOA.

	Erosive (n = 303)	Non-erosive (n = 736)		
	Mean (SD)	Mean (SD)	OR (95%CI)	*p*
Age at diagnosis	59 (8.17)	65 (8.97)	0.93 (0.92–0.95)	**< 0.001**
BMI	30 (12.96)	29 (24.61)	1.00 (0.98–1.01)	0.653
Abdominal perimeter (cm)	93 (12.71)	96 (11.98)	0.98 (0.97–0.99)	**0.002**
Total AUSCAN	54 (26.92)	41 (29.50)	1.02 (1.01–1.02)	**< 0.001**
Pain AUSCAN	56 (27.81)	43 (31.85)	1.01 (1.01–1.02)	**< 0.001**
Stiffness AUSCAN	54 (32.88)	39 (36.00)	1.01 (1.01–1.02)	**< 0.001**
Function AUSCAN	54 (28.44)	40 (30.28)	1.02 (1.01–1.02)	**< 0.001**
ESR	20 (13.41)	19 (14.91)	1 (0.99–1.01)	0.408
CRP (mg/dL)	0 (0.54)	0 (1.01)	0.95 (0.75–1.12)	0.597
	**n (%)**	**n (%)**		
Sex				
Male	38 (12.54)	143 (19.43)	0.6 (0.40–0.87)	**0.009**
Female	265 (87.46)	593 (80.57)	1.00	
Hypertension	114 (37.75)	385 (52.52)	0.55 (0.41–0.72)	**< 0.001**
Diabetes	57 (18.87)	129 (17.67)	1.09 (0.76–1.53)	0.641
Dyslipidemia	168 (55.45)	431 (58.72)	0.88 (0.67–1.15)	0.344
Hypertriglyceridemia	47 (15.72)	121 (17.26)	1.09 (0,76–1.53)	0.550
Low HDL	30 (9.97)	65 (9.25)	0.88 (0.67–1.15)	0.344
MetS	50 (16.50)	152 (20.82)	0.75 (0.53–1.07)	0.114
Osteoporosis	44 (14.52)	122 (16.58)	1.33 (0.93–1.88)	0.116
Osteopenia	30 (9.90)	77 (10.46)	0.86 (0.58–1.24)	0.417
Smoking habit				
Never	177 (58.42)	482 (65.94)	1.00	
Current	39 (12.87)	83 (11.35)	1.28 (0.84–1.94)	0.243
Former	87 (28.71)	166 (22.71)	1.43 (1.05–1.95)	**0.024**
Psoriasis	21 (6.93)	66 (9.02)	0.9 (0.62–1.29)	0.557
Knee OA (KL > I)	155 (53.26)	558 (77.50)	0.33 (0.25–0.44)	**< 0.001**
Hip OA	109 (37.33)	406 (57.43)	0.44 (0.33–0.58)	**< 0.001**
Inflammatory hand OA	80 (26.49)	37 (5.08)	6.74 (4.44–10.24)	**< 0.001**
Nodular hand OA	290 (96.03)	566 (78.72)	6.53 (3.57–11.95)	**< 0.001**
Thumb base OA	160 (53.16)	489 (67.45)	0.55 (0.42–0.72)	**< 0.001**
Positive RF	24 (8.42)	57 (8.77)	0.96 (0.58–1.57)	0.856
Positive ACPA	3 (1.11)	9 (1.52)	0.72 (0.19–2070)	0.630

BMI, body mass index (Kg/m^2^); HDL, high-density lipoprotein; MetS, metabolic syndrome; Knee OA, OA at least in one knee; Hip OA, OA at least in one hip; EHOA, erosive hand osteoarthritis at least in one hand; Inflammatory hand OA, OA at least in one hand; Nodular hand OA, OA at least in one hand; Thumb base OA, OA at least in one hand; ESR, erythrocyte sedimentation rate (mmHg); CRP, C-reactive protein (mg/dL); RF, rheumatoid factor; ACPA, anti-citrullinated protein antibodies.

Significant values are in [bold].

Comparing patients with EHOA to non-EHOA, there was a lower representation of hypertension (37.75 vs. 52.52%; *p* < 0.001), knee OA (53.26 vs. 77.5%; *p* < 0.001), hip OA (37.33 vs. 57.43%; *p* < 0.001), and thumb base OA (53.16 vs. 67.45%; *p* < 0.001) (Table 2). Conversely, the EHOA group exhibited a higher presence of clinically inflamed joints (26.49 vs. 5.08%; *p* < 0.001) and nodular hand OA (96.03 vs. 78.72%; *p* < 0.001).

**Figure 1:**
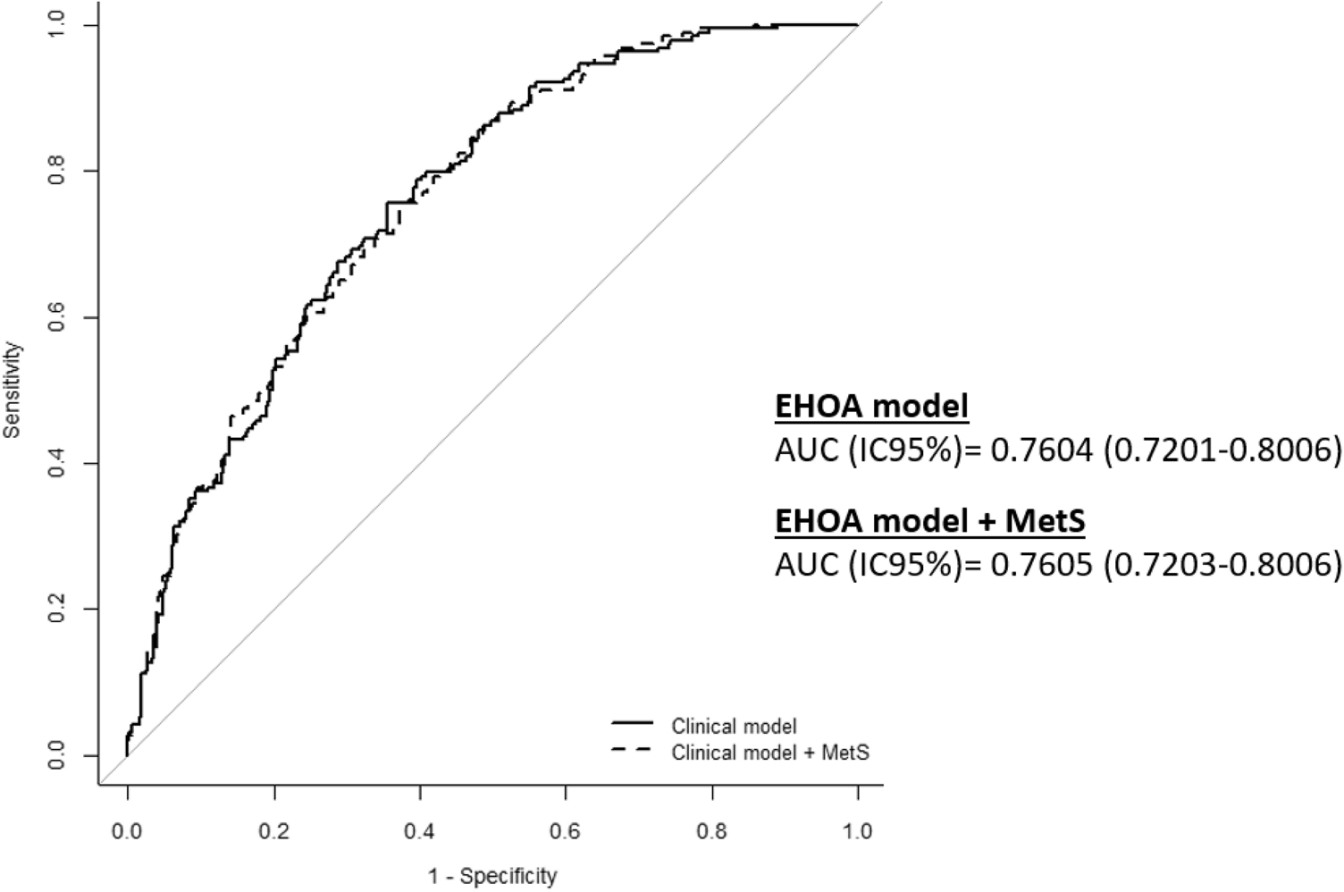
ROC curve of EHOA predictive model without and with MetS The continuous line represents the data from the EHOA predictive model that includes the variables from Tables 3 and 4 (age at diagnosis, sex, hypertension, body mass index, knee OA, nodular hand OA, inflammatory hand OA and hip OA). This model has a sensitivity of 0.75, an specificity of 0.64, a negative predictive value of 0.83 and a positive predictive value of 0.53. The dashed line represents the model by adding metabolic syndrome (MetS). This model with MetS has a sensitivity of 0.75, an specificity of 0.63, a negative predictive value of 0.83 and a positive predictive value of 0.51.

The most significant factors independently associated with EHOA, after adjusting for variables (age, sex, hypertension, hip OA, and BMI), were the concurrent presence of inflamed joints (OR = 3.21; 95%CI 1.86–5.52; *p* < 0.001), nodular hand OA (OR = 7.47; 95%CI 2.89–19.31; *p* < 0.001), and total AUSCAN (OR = 1.01; 95%CI 1.01–1.21; *p* < 0.001). Additionally, the association with a lower prevalence of knee OA (OR = 0.57; 95%CI 0.35–0.92; *p* = 0.021) remained significant (Table 3). This EHOA predictive model exhibited an AUC of 0.75, sensitivity of 0.75, and specificity of 0.64 (Table 3 and Fig. 1).

**Table 3:** Erosive hand OA predictive model.

Model	B	EE	*p*	OR	95% CI (OR)	
Age at diagnosis	− 0.019	0.013	0.146	0.98	0.95	1.00
Sex (Male vs Female)	0.566	0.313	0.071	1.76	0.95	3.25
Hypertension	− 0.341	0.217	0.117	0.71	0.46	1.08
BMI (basal)	0.003	0.004	0.48	1.00	0.99	1.01
Knee OA (KL > I)	− 0.566	0.246	**0**.**021**	**0**.**57**	0.35	0.91
Nodular Hand OA	2.011	0.484	**< 0**.**001**	**7**.**47**	2.89	19.31
Immflamatory Hand OA	1.165	0.277	**< 0**.**001**	**3**.**21**	1.86	5.52
Hip OA	− 0.21	0.219	0.338	0.81	0.52	1.24
Total AUSCAN (basal)	0.013	0.004	**< 0**.**001**	**1**.**01**	1.00	1.02
Constant	− 1.856	0.933	0.047	0.16		
AUC (IC95%) = 0.76 (0.72–0.80)						

Significant values are in [bold].

Regarding MetS, only hypertension and abdominal circumference showed a statistically significant relationship in univariate analysis, but these results were not sustained in multivariate analysis (Table 3).

In analyzing the influence of MetS on EHOA patients by including MetS as a covariate in the proposed model, it was observed that MetS did not significantly influence the presence of EHOA, maintaining the size of the effect of other factors (Table 4). Although not an independent factor for predicting EHOA in the analyzed sample, a non-statistically significant trend of increased EHOA risk was observed among patients with MetS (OR = 1.54; 95%CI 0.89–2.68) (Table 4 and Fig. 1).

**Table 4:** Erosive Hand OA predictive model including MetS.

Model + MetS	B	EE	*p*	OR	95% CI (OR)	
Age at diagnosis	− 0.022	0.014	0.104	0.97	0.95	1.00
Sex	0.486	0.317	0.125	1.62	0.87	3.02
Hypertension	− 0.461	0.233	**0**.**048**	0.63	0.39	0.99
BMI	0.003	0.004	0.491	1.00	0.99	1.01
Knee OA (KL > I)	− 0.591	0.247	**0**.**017**	0.55	0.34	0.89
Nodular Hand OA	1.96	0.485	**< 0**.**001**	7.1	2.74	18.36
Immflamatory Hand OA	1.175	0.278	** < 0**.**001**	3.23	1.87	5.58
Hip OA	− 0.232	0.22	0.29	0.79	0.51	1.21
Total AUSCAN (basal)	0.013	0.004	**< 0**.**001**	1.01	1.00	1.02
MetS	0.433	0.282	**0**.**125**	**1**.**54**	0.88	2.68
Constant	− 1.675	0.941	0.075	0.18		
AUC (IC95%) = 0.76 (0.72–0.80)						

BMI, body mass index (Kg/m^2^); Knee OA, OA at least in one knee; Nodular hand OA, OA at least in one hand; Inflammatory hand OA, OA at least in one hand; Hip OA, OA at least in one hip; MetS, metabolic syndrome.

Significant values are in [bold].

**Figure 2:**
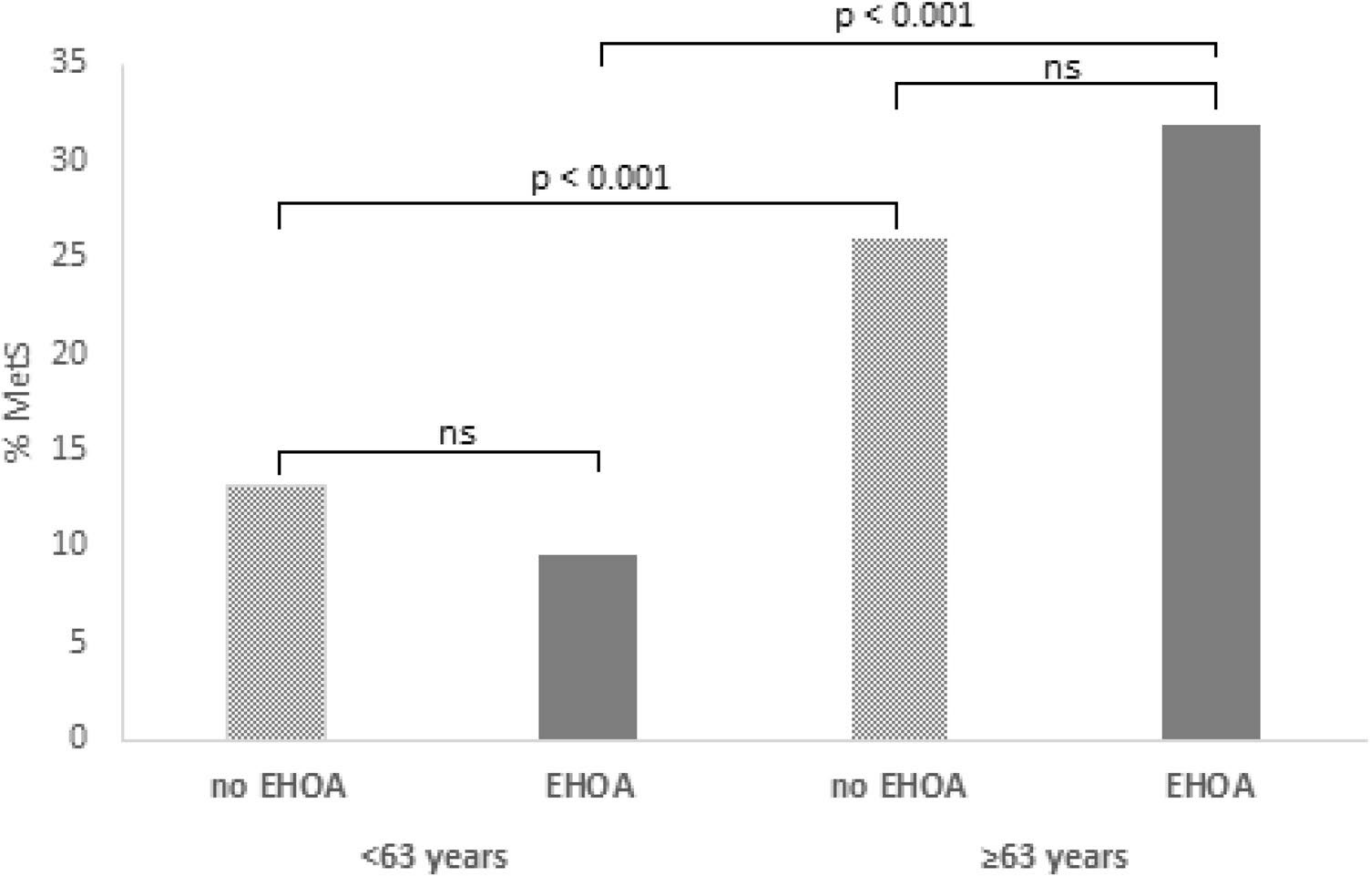
Correlation between age and metabolic syndrome (MetS) in patients with and without Erosive Hand OA (EHOA).

Moreover, multivariable-adjusted restricted cubic splines demonstrated a non-significant relationship between age and EHOA (p for overall trend = 0.335) (Supplementary Fig. 1). Upon thorough data review and considering a threshold at age 63 to explore a possible change in the association between age and EHOA risk and its interaction with MetS, a reversed trend was observed among those over 63, indicating a higher prevalence of MetS among EHOA (31.91 vs. 26.04%), although these differences were not significant in either group (Supplementary Table 1 and Fig. 2).

Analyzing the association between age and MetS independently revealed a significant association, with a higher prevalence of metabolic syndrome in those over 63 years of age (Fig. 2). Among patients ≥ 63 years, there were more with non-EHOA than with EHOA, while EHOA predominated among the youngest. Analyzing age and MetS within each erosive group (non-EHOA vs. EHOA) showed a significant association between age and MetS in both groups, with a greater presence of MetS among those ≥ 63 years of age (Fig. 2).

The number of erosions, total erosions on both hands, and the average number of erosions between the hands were recorded. The mean number of total erosions between both hands was around 3, both in patients with MetS and without MetS (*p* = 0.327) (Table 5).

**Table 5:** Relation between number of erosions in hand OA and MetS.

Total number of erosions in both hands								
Metabolic syndrome	n	Mean	SD	Mediane	Min	Max	Q1	Q3
No	208	3.33	2.36	3	0	13	2	5
Yes	43	3.88	2.89	4	0	11	1	6
*p* = 0.327								

## Discussion

One objective of this study was to describe the Erosive Hand Osteoarthritis (EHOA) phenotype in the Spanish PROCOAC cohort. Characterizing this phenotype revealed that, compared to non-EHOA, patients with EHOA are younger, exhibit a lower presence of knee Osteoarthritis (OA), a higher concurrent presence of clinically inflamed joints, and an increased prevalence of nodular hand OA.

The heightened levels of pain, disability, and inflammation observed in EHOA patients, in addition to the increased presence of nodes and erosions, contribute to a greater clinical burden when compared to patients with non-erosive hand OA, aligning with prior findings^7,22,38,39^.

In our cohort, EHOA showed no association with Rheumatoid Factor (RF). These results are consistent with previous publications^40–42^, and similarly, there is no observed relationship between EHOA and Anti-Cyclic Citrullinated Peptide antibodies (ACPA), a novel finding not reported before in our knowledge. However, there are existing data about the connection between ACPA and inflammatory hand OA^43^.

The second aim was to elucidate the relationship between EHOA and Metabolic Syndrome (MetS). Univariate and multivariate analyses were conducted without revealing a significant association. Additionally, a predictive model for EHOA incorporating MetS showed no modification in the results. This is in concordance with findings from the Strand publication based on the Framingham cohort, which found no significant association between hand OA, including EHOA, and MetS^44^. Notably, there was a borderline association with hypertension, aligning with our cohort data.

The association of MetS with hand OA and EHOA has yielded conflicting results in the literature. While some studies suggest a potential link, others present contradictory evidence^25,26,27^. Our study adds to this complexity, emphasizing the need for further investigation. Notably, our analysis of EHOA patients did not demonstrate a significant association with MetS, consistent with findings from the Framingham cohort^44^.

The association of hand OA or EHOA with more generalized OA has been debated^5,21,45^. Our study indicates a lower prevalence of OA in other joints, specifically the knees, among patients with EHOA in the PROCOAC cohort. This could be attributed to the relatively lower age of EHOA patients and their lower BMI compared to non-EHOA patients. However, this finding contrasts with some studies showing a more common occurrence of hand OA in the context of generalized OA^20^. A recent publication shows the inverse associationof two loci between EHOA and knee OA^46^. In this paper they found 4 significant EHOA loci with high effect on EHOA risk. Two of them, rs1800801 and rs4496445 was associated with EHOA risk and with protection of knee OA. Apart from the manuscript of Haugen^18^ and Marshall^13^, to our knowledge, none of the abrove-mentioned studies focused their analysses on the erosive subtype specifically, but rahter on hand OA in general. The study of Marshall et al. consisted of the description of subsets of hand OA in a prospective cohort of 1076 older adult patients, of which only 52 (4.8%) developed the erosive phenotype and they concluded that radiographic knee OA was not increased in EHOA patients.

While the present study focuses on EHOA, the analysis of other studies is predominantly centered on hand OA in general. Future research with large, prospective cohorts is imperative to delve deeper into the association between EHOA and other joint involvements.

This study has potential limitations, including its cross-sectional approach to hand OA patients, limiting the establishment of causality. Unmeasured confounders, such as diet, alcohol consumption, or socioeconomic status, could influence the results. Nevertheless, the proportion of EHOA in our cohort, at 20.3%, exceeds the mean compared with other studies. Other studies patients.

## Conclusion

Within the PROCOAC cohort, Erosive Hand Osteoarthritis (EHOA) demonstrates associations with nodular hand OA, inflammatory hand OA, and elevated total AUSCAN scores. Notably, EHOA exhibits a lower prevalence of knee Osteoarthritis (OA) in our cohort. Additionally, we observed no discernible relationship between EHOA and Metabolic Syndrome (MetS) in our study.

### Supplementary Information

Supplementary Information.

## Data Availability

The datasets used and/or analysed during the current study available from the corresponding author on reasonable request.
