# Supplementary material for: Metabolic syndrome is not associated with erosive hand osteoarthritis: a cross-sectional study using data from the PROCOAC cohort
Source: Sci Rep. 2024 Mar 12;14:5968. doi: 10.1038/s41598-024-55374-1 (PMC10933413; doi:10.1038/s41598-024-55374-1)
Supplement: Supplementary file 1 — Supplementary Information. [file 41598_2024_55374_MOESM1_ESM.docx]

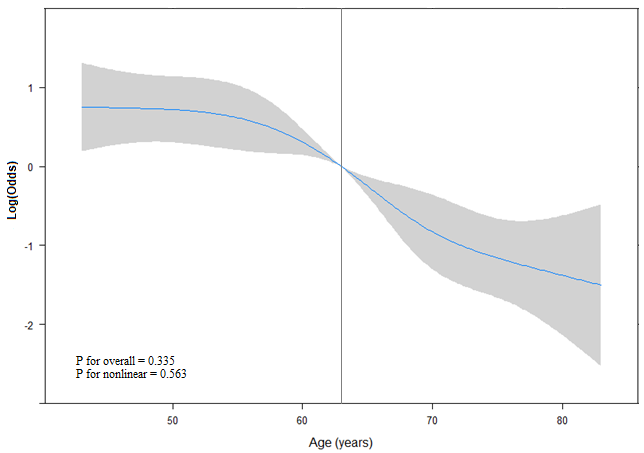


**Supplementary Figure 1:** Restricted cubic splines curve for EHOA by age after covariate adjustment. Central line represents the estimated log Odds, with shaded ribbons denoting 95% confidence interval. The vertical line indicates the threshold value of age at 63 years. The model is adjusted for sex, hypertension, body mass index, knee OA, nodular hand OA, inflammatory hand OA, Hip OA, total AUSCAN and MetS.

**Supplementary Table 1: Correlation between age and metabolic syndrome (MetS) in patients with and without Erosive Hand OA (EHOA)**

|  |  |  |  |  | **Patients with MetS** | |  |
| --- | --- | --- | --- | --- | --- | --- | --- |
|  | **No MetS** | **MetS** | **No EHOA** | **EHOA** | **No EHOA** | **EHOA** |  |
|  | **n (%)** | **n (%)** | **n (%)** | **n (%)** | **n (%)** | **n (%)** | **p** |
| **<63 years** | 457 (52,8) | 64 (25,1) | 297 (40,4) | 209 (69,0) | 39 (13,18) | 20 (9,57) | 0,214 |
| **≥63 years** | 409 (47,2) | 191 (74,9) | 439 (59,6) | 94 (31,0) | 113 (26,04) | 30 (31,91) | 0,245 |
| **P value** | **<0,001** |  | **<0,001** |  | **<0,001** | **<0,001** |  |
